# Higher body mass index is associated with episodic memory deficits in young adults

**DOI:** 10.1080/17470218.2015.1099163

**Published:** 2016-02-22

**Authors:** Lucy G. Cheke, Jon S. Simons, Nicola S. Clayton

**Affiliations:** ^a^ Department of Psychology, University of Cambridge, Cambridge, UK

**Keywords:** Obesity, Memory, Appetite regulation, Episodic memory, What–where–when

## Abstract

Obesity has become an international health crisis. There is accumulating evidence that excess bodyweight is associated with changes to the structure and function of the brain and with a number of cognitive deficits. In particular, research suggests that obesity is associated with hippocampal and frontal lobe dysfunction, which would be predicted to impact memory. However, evidence for such memory impairment is currently limited. We hypothesised that higher body mass index (BMI) would be associated with reduced performance on a test of episodic memory that assesses not only content, but also context and feature integration. A total of 50 participants aged 18–35 years, with BMIs ranging from 18 to 51, were tested on a novel what–where–when style episodic memory test: the “Treasure-Hunt Task”. This test requires recollection of object, location, and temporal order information within the same paradigm, as well as testing the ability to integrate these features into a single event recollection. Higher BMI was associated with significantly lower performance on the what–where–when (WWW) memory task and all individual elements: object identification, location memory, and temporal order memory. After controlling for age, sex, and years in education, the effect of BMI on the individual what, where, and when tasks remained, while the WWW dropped below significance. This finding of episodic memory deficits in obesity is of concern given the emerging evidence for a role for episodic cognition in appetite regulation.

Obesity has become one the of most significant health concerns facing the western world. In the United Kingdom, around 65% of adults are overweight, and 25% are obese (World Health Organization, 2010). Obesity is a major risk factor for premature mortality (Kopelman, 2000) and carries an enormous financial burden for governments and health care providers worldwide. As such, research into understanding how this problem perpetuates is of high priority.

While the physical health impacts of obesity are increasingly well understood, recent research indicates that there may be a significant psychological element to the obese syndrome, with proposals that cognitive deficits may occur both as a result of obesity and potentially as a causal factor in its emergence.

Accumulating evidence suggests that obesity-related health issues (such as diabetes and hypertension) along with adiposity (excess body weight) itself have a significant impact on the structure and function of the brain both in rodent models (e.g., Erion et al., 2014; Kanoski, Meisel, Mullins, & Davidson, 2007; Molteni, Barnard, Ying, Roberts, & Gomez-Pinilla, 2002) and in humans (Bruehl, Sweat, Tirsi, Shah, & Convit, 2011; Jagust, Harvey, Mungas, & Haan, 2005; Mueller et al., 2012; Raji et al., 2010; Smith et al., 2015; Ursache, Wedin, Tirsi, & Convit, 2012). These neurological changes are accompanied by evidence of cognitive deficits (e.g., Reinert, Po'e, & Barkin, 2013). Because of the central role of behaviour in the advancement of the obese syndrome through high consumption and low energy expenditure, evidence for obesity-related cognitive change has inspired a number of addiction (see Smith & Robbins, 2013) and “vicious cycle” (Kanoski & Davidson, 2011; Sellbom & Gunstad, 2012) models of obesity that describe a circular pattern of obesity, behavioural change, and consumption.

Episodic memory is the ability to store, maintain, and retrieve contextually rich representations of events from one's own life (Tulving & Donaldson, 1972). There is increasing evidence to suggest that this type of memory may play a major role in allowing us to regulate consumption. Manipulations of memory for recent meals have considerable impact on the long-term satiating effect of those meals (e.g., Brunstrom et al., 2012; Higgs & Donohoe, 2011; Higgs, Williamson, & Attwood, 2008; Oldham-Cooper, Hardman, Nicoll, Rogers, & Brunstrom, 2011), while amnesic patients who are unable to remember recent consumption can sometimes eat several consecutive meals without reporting satiety or discomfort (Hebben, Corkin, Eichenbaum, & Shedlack, 1985; Higgs, Williamson, Rotshtein, & Humphreys, 2008; Rozin, Dow, Moscovitch, & Rajaram, 1998). Rodent models suggest that neurotoxic lesions to the hippocampus analogous to those seen in episodic amnesia result in animals that will work harder for food (e.g., Clifton, Vickers, & Somerville, 1998) and are more likely to become overweight (Davidson et al., 2009) than sham-lesioned controls. While studies involving humans and animals with severe brain lesions are difficult to generalize to the general population, this cumulative evidence suggests that episodic memory plays a significant role in the regulation of consumption, and that damage to brain areas associated with memory such as the hippocampus may result in overconsumption and, at least in rodent models, obesity. However, homeostasis is a complex process, and it is clear that other cognitive factors such as executive functions and Pavlovian learning (Davidson, Tracy, Schier, & Swithers, 2014) are highly involved in the control of food intake and may also be disrupted by temporal lobe lesions (Davidson et al., 2010). To date there is no research investigating the impact of minor memory deficits on consumption. However, the findings of Higgs and colleagues suggest that small individual differences in memory accuracy or vividness may be capable of having a considerable influence. It is therefore important to establish whether obesity is associated with episodic memory deficits.

There is accumulating evidence that obesity and obesity-related health disorders may be a contributing factor to changes to areas within the “core recollection network” of the brain (Rugg & Vilberg, 2013), and in particular to hippocampal structure and function. Rodent models have produced extensive evidence for changes in hippocampal structure and function in obese animals (Grillo et al., 2011; Li et al., 2002; Molteni et al., 2002), suggesting that both dietary and congenital obesity lead to abnormalities in the hippocampal formation. Recent studies are beginning to show a similar pattern of neural change in humans (Gustafson, Lissner, Bengtsson, Bjorkelund, & Skoog, 2004; Raji et al., 2010; Ursache et al., 2012).

There have been a number of mechanisms proposed as potential drivers of the hippocampal changes seen in obesity. Given the association between obesity and type 2 diabetes mellitus (Bonadonna et al., 1990), insulin resistance has been suggested as a key mechanism in hippocampal dysfunction in obesity (Chabot, Massicotte, Milot, Trudeau, & Gagne, 1997; Lamport, Lawton, Mansfield, Moulin, & Dye, 2014; Li et al., 2002; Molteni et al., 2002; Zhao, Chen, Quon, & Alkon, 2004). Indeed, obese individuals with diabetes show hippocampal volumetric reductions (Bruehl et al., 2011; Ursache et al., 2012) and impaired source memory performance (Lamport et al., 2014) compared to their nondiabetic obese peers. However, given the evidence for hippocampal volume reductions in obesity in the absence of diabetes (Jagust et al., 2005; Raji et al., 2010), it is likely that the effect of insulin resistance is additive rather than explanatory. Indeed, adiposity itself is associated with neuroinflammation (Erion et al., 2014), suggesting that being overweight may be sufficient to cause alterations to brain function, independent of how obesity was achieved and what comorbid health problems may exist. Nonetheless, given the high levels of comorbidity of obesity with health issues that, like diabetes, have been associated with cognitive decline (e.g., hypertension, Kilander, Nyman, Boberg, Hansson, & Lithell, 1998; and sleep apnoea, Décary, Rouleau, & Montplaisir, 2000), it may be constructive to address cognitive deficits in the obese “syndrome”, considering not just adiposity but the combination of disorders that often accompany it.

Hippocampal abnormalities seen in rodent models of obesity are associated with robust evidence for memory and spatial cognition deficits in these animals (Jurdak, Lichtenstein, & Kanarek, 2008; Molteni et al., 2002; Popovic, Biessels, Isaacson, & Gispen, 2001; Valladolid-Acebes et al., 2011; Winocur et al., 2005). However, the association between obesity and memory in humans is much less clear. Obese adults have been reported to perform poorly on measures of verbal learning such as delayed recall and recognition (Cournot et al., 2006; Elias, Elias, Sullivan, Wolf, & D'Agostino, 2003; Gunstad, Paul, Cohen, Tate, & Gordon, 2006), an effect that is independent from, but interacts with, the effects of normal ageing (Gunstad, Lhotsky, Wendell, Ferrucci, & Zonderman, 2010). However, other studies have failed to find evidence for obesity-related memory impairments (Conforto & Gershman, 1985; Holloway et al., 2011; Nilsson & Nilsson, 2009).

This inconsistent picture may be due to methodological issues. So far, memory in obese subjects has only been investigated in the context of verbal recall of word lists, a task that may have little in common with the contextually rich multidimensional episodic recollection that has been linked with consumption. The current study aimed to investigate whether overweight individuals are impaired in memory for complex temporal–spatial events—that is, whether they are less able to recall the *what, where, and when* (WWW) elements of an episode. Such features are considered to be definitive of episodic memory (Tulving & Donaldson, 1972) and have been extensively used to assess episodic memory behaviourally in nonhuman animals (e.g., Babb & Crystal, 2006; Clayton & Dickinson, 1998). Recent studies in healthy human participants have shown that WWW memories are reliably reported as “remembered” rather than “known” (Easton, Webster, & Eacott, 2012; Holland & Smulders, 2011), indicating a strong dependence on recollection. Furthermore, integrated WWW memories have been shown to be impaired in normal ageing, and related to memory complaints in older adults, to a greater degree than retrieval of the individual what, where, and when elements, or free recall performance (Plancher, Gyselinck, Nicolas, & Piolino, 2010). WWW performance has been shown to be correlated with, but distinct from, free recall performance (Cheke & Clayton, 2013, 2015) motivating the concept that while both are tests of episodic memory, they may be assessing different aspects of this ability. Evidence from rodent models (sometimes using variants in which the “when” element is replaced with “which context”) suggests that WWW memories rely on the integrity of the hippocampus (DeVito & Eichenbaum, 2010; Ergorul & Eichenbaum, 2004). Crucially, it appears that integrated WWW memories are dependent on hippocampal function, whereas the component elements (what, what–where, when/which context) are preserved despite hippocampal lesions (Langston & Wood, 2010). WWW memory and spatial memory (but not object memory alone) are also shown to be sensitive to normal ageing and Alzheimer's-like pathology in mice (Davis, Eacott, Easton, & Gigg, 2013). Thus it appears to be the spatial component, and in particular the requirement for integration of multiple types of information, that depends on intact hippocampal function. While most of this research has concentrated on the hippocampus, it is likely that WWW tests require the healthy function of a range of areas within the “core recollection network”, including the prefrontal and parietal cortices (Rugg & Vilberg, 2013).

Here we introduce the “Treasure-Hunt Task”. This task assesses memory for object information (“what”), location information (“where”), and temporal order information (“when”) within the same paradigm, as well as testing the ability to integrate these features into a single “WWW” event recollection. In this way, this task is able to identify not just the extent but the *pattern* of performance deficits that may characterize different disorders.

## EXPERIMENTAL STUDY

### Ethics statement

This study was approved by the Cambridge Human Biology Research Ethics Committee. All participants gave written informed consent to take part.

### Method

A total of 60 participants between 18 and 35 years were recruited through posters, online advertisements, and word of mouth. A high proportion of the first participants to be recruited indicated on a demographic questionnaire that they had a history of mental illness, on the basis of which the data of these individuals (*n* = 10) were excluded prior to analysis. Later recruitment screened participants for a history of mental illness before arrival. This left 50 participants (72% female; mean age 24.62 years, range 18–35 years; mean body mass index [BMI] 25.7, range 18–51.7). Of these, 26 were lean (BMI < 25), and 24 were overweight (*n* = 16, BMI = 25–30) or obese (*n* = 8, BMI > 30). Recruitment across the BMI range was balanced for recruitment population (e.g., university/nonuniversity) and age. All participants indicated that they had never received a diagnosis of diabetes.

**Figure 1:**
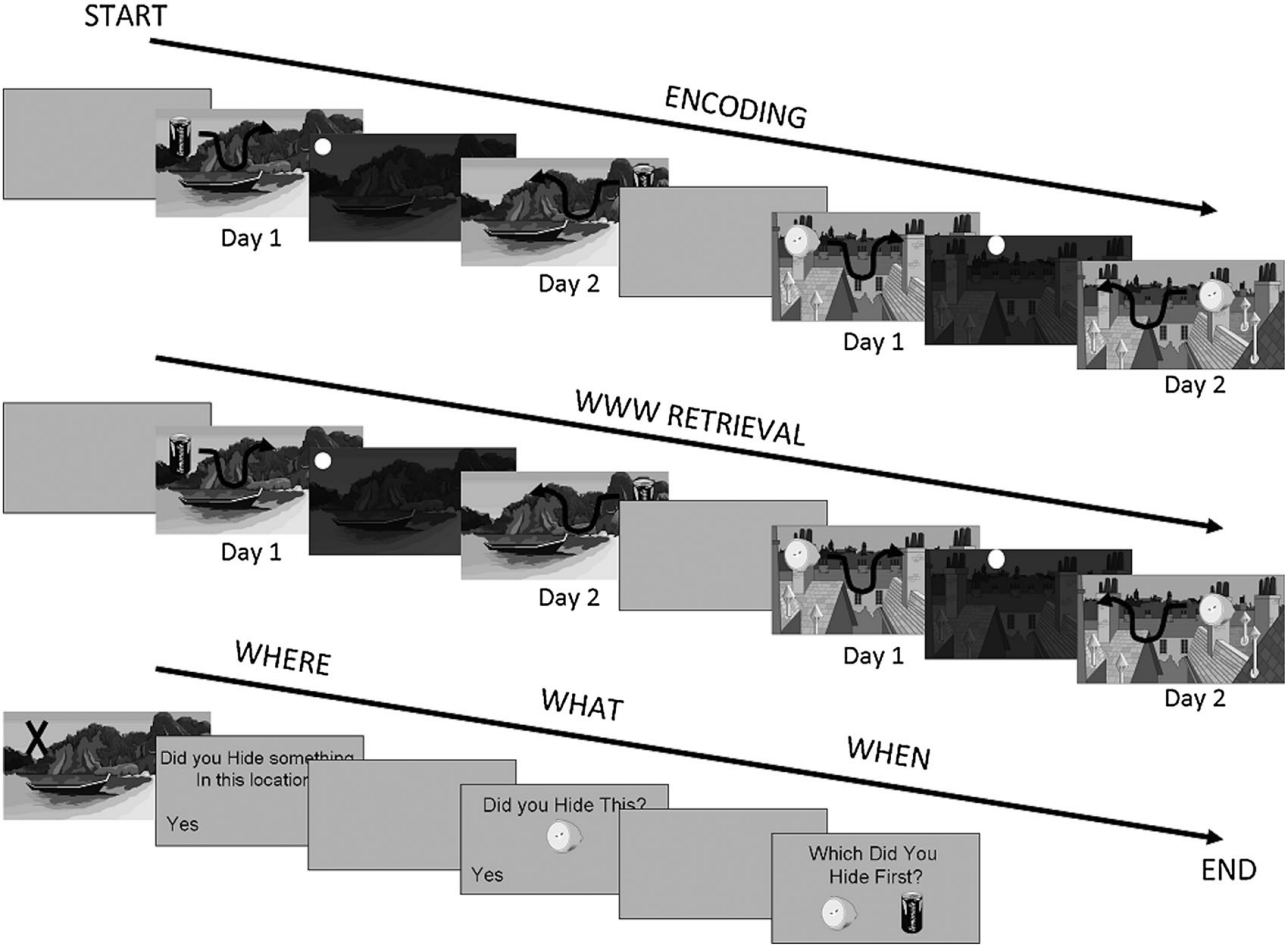
Schematic of the memory test. Participants moved items around and “hid” them in two scenes across two “days” (“encoding”). Participants were then asked to indicate in the same manner where they had hidden each food on each day (“WWW retrieval”, where WWW = what–where–when). They were then given the “where” and “what” recognition tests, followed by the “when” order discrimination test.

Participants were invited to attend a testing session in the Department of Psychology in the centre of Cambridge. Here they completed a demographic information form and a training task. They then undertook six sessions of the computerized memory task created using PsychoPy (Peirce, 2008). The memory task contained five sections: encoding, WWW, where, what, and when (see Figure 1). These were presented in a fixed order. During the encoding period, participants were instructed to move a number of food items around a complex scene (for example, a desert with palm trees; see Supplemental Material for examples of scenes and items) using the arrow keys, and to “hide” them in the scene by pressing enter. Participants were specifically instructed to use the “scene, not the screen” and not to hide in the corners of the screen. Each item was hidden within a given scene twice, across two hiding periods labelled “day 1” and “day 2”, which occurred consecutively. Within each encoding period, participants hid objects in two different scenes consecutively (such that the order was: Scene 1, Day 1; Scene 1, Day 2; Scene 2, Day 1; Scene 2, Day 2). The WWW retrieval period occurred immediately after encoding; however, because memory for Scene 1 was always assessed first, the encoding of Scene 2 occurred during the retention interval for Scene 1, and retrieval of Scene 1 occurred during the retention interval for Scene 2, meaning that the retention interval was around 5 min. During the WWW retrieval period, participants were instructed to move each of the food items around the screen just as they had during encoding, but this time in order to indicate where they hid that item in that scene on each “day” (“place the item in the same place you hid it on day 1”). This was followed by the “where” retrieval period during which participants observed a series of “X”s in specific locations within the scenes for 5 seconds. After each, they were asked “Did you hide something in that location?”. Half of the “X”s were in a location where the participant had hidden an item; half were in locations in which the participant had not hidden anything. The participants were then shown a series of food items, half of which they had hidden and half of which were new. They were asked “Did you hide this item?” (“what” retrieval period). Finally, subjects were shown two items and were asked “which of these did you hide first?” (“when” retrieval period). This allowed participants to be tested on the order of appearance of items within as well as between scenes, facilitating a greater range of questions. As such, the last item from Scene 1 would be considered to have appeared before the first item of Scene 2, but after the first item of Scene 1. While each item appeared on both “Day 1” and “Day 2” in each scene, the participants were asked to consider when they *first* hid that item. There were six different sessions of these tasks, divided into two “easy” sessions (with four items, and therefore eight hiding events since each was hidden twice), two “medium” sessions (with eight items, and therefore 16 hiding events), and two “hard” sessions (with 12 items and 24 hiding events). Thus each retrieval task had eight items in the easy sessions, 16 in the medium sessions, and 24 in the hard sessions, with the exception of the easy “when” tasks, which had only six items because that is the maximum number of permutations for four items. Participants undertook these six sessions in a random order, counterbalanced across individuals.

Accuracy on the “WWW” task was calculated as proportion of items in which the participant indicated the correct location. Responses on this task were also coded for the types of errors made. “Imprecision” errors were defined as those responses that were within one key-press of the correct location, but did not match this location completely. This type of error suggests that the correct what–where–when combination has been retrieved, but imprecisely reported. “Binding” errors were defined as those where the correct location had been reported, but for the wrong object, the right object on the wrong day, or the wrong object on the wrong day. Finally “total inaccuracy” errors were defined as those that did not match any features with any correct location. It is difficult to know exactly what failure causes such errors as they could result either from total binding failures or from total failures at spatial memory.

Accuracy on the “where” and “what” tasks was computed by calculating *d* ′ from proportion of correctly identified “old” items/locations against false alarms (new items identified as old). Accuracy for the “when” task was computed by calculating *d* ′ from proportion of correct answers against proportion of incorrect answers. Formulas for *d* ′ calculation were taken from Macmillan and Creelman (Macmillan & Creelman, 1990). Analysis was conducted using repeated measures analysis of variance (ANOVA) with difficulty as a between-subjects factor and BMI as a covariate, and stepwise linear regression.

Internal consistency for all tests was very high (Cronbach's alpha, WWW: *α* = .898; “what”: *α* = .896; “where”: *α* = .74; “when”: *α* = .719].

### Results

There were no differences between men and women on any of the memory measures [“WWW”: *t*(48) = 1.405, *p* = .166; “what”: *t*(48) = 0.237, *p* = .814; “where”: *t*(48) = 1.438, *p* = .157; “when”: *t*(48) = 0.079, *p* = .937]. The data from both sexes were pooled for all subsequent analyses.

**Figure 2:**
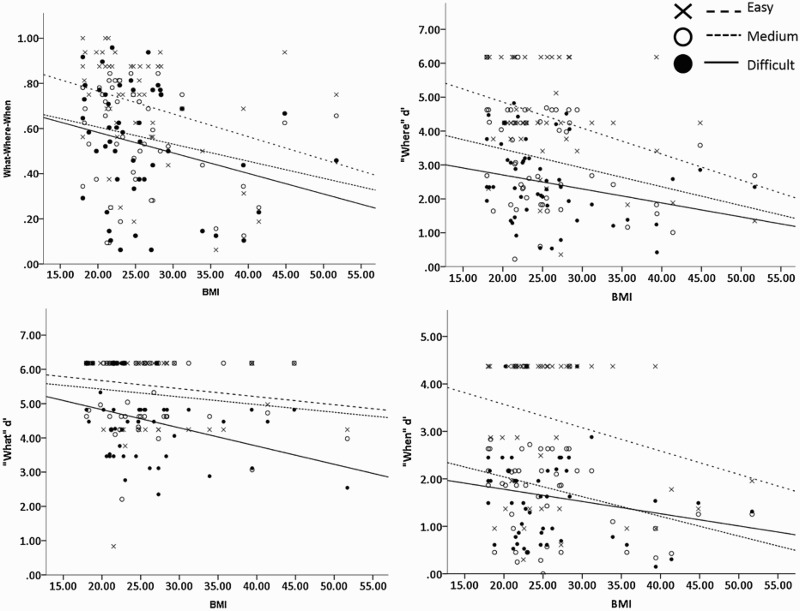
Association between memory score and body mass index (BMI) in what–where–when (*r* = −.295; top left), where (*r* = −.346; top right), what (*r* = −.228; bottom left), and when (*r* = −.272; bottom right) tasks.

There was a significant negative effect of increased BMI on performance on all tasks [“WWW”: *F*(1, 48) = 4.567, *p* = .038; “where”: *F*(1, 48) = 9.696, *p* = .003; “what”: *F*(1, 48) = 5.758, *p* = .02; “when”: *F*(1, 48) = 5.181, *p* = .027]. When controlling for BMI, performance did not significantly reduce with increasing difficulty levels on the WWW or what tasks [“WWW”: *F*(2, 47) = 3.082, *p* = .055; “what”: *F*(2, 47) = 0.093, *p* = .911] but did on the “where”, *F*(2, 47) = 4.648, *p* = .014, and “when” tasks, *F*(2, 47) = 3.789, *p* = .03. In no task was there an interaction between BMI and difficulty level [“WWW”: *F*(2, 47) = 0.242, *p* = .786; “what”: *F*(2, 47) = 1.057, *p* = .355; “where”: *F*(2, 47) = 0.523, *p* = .596; “when”: *F*(2, 47) = 0.462, *p* = .633]. These results suggest that with increasing BMI, individuals struggled with all aspects of the Treasure-Hunt Task, but did not become more impaired as the difficulty increased (Figure 2).

In our sample, BMI was significantly related to years in education, *r*(50) = −.374, *p* = .008, and gender, *t*(48) = 2.779, *p* = .008, but not age, *r*(50) = 0.212, *p* = .139. It is therefore important to establish the additional variance in memory score explained by BMI beyond that explained by years in education and gender. When entered into a stepwise regression analysis with age, gender, and years in education, BMI explained an additional 6.1% of variance in WWW score, which was not a significant change from the variance explained by age, gender, and years in education alone (*p* = .085). However, BMI did account for significant additional variance in performance on the “what”, *r* ^2^ change = .094, *p* = .035, “where”, *r* ^2^ change = .239, *p* < .001, and “when”, *r* ^2^ change = .096, *p* = .03, tasks. On their own, age, years in education and gender predicted very little variance in memory performance, and in no test did the influence of these factors differ significantly from zero [WWW: *r* ^2^ = .06, *p* = .414; “where”: *r* ^2^ = .059, *p* = .422; “when”: *r* ^2^ = .044, *p* = .558; “what”: *r* ^2^ = .010, *p* = .923].

The effect of BMI on memory performance was not reflected in reaction times. There was no relationship between BMI and reaction times on any task [“WWW”: *F*(1, 48) = 0.071, *p* = .970; “what”: *F*(1, 48) = 0.101, *p* = .752; “where”: *F*(1) = 0.094, *p* = .760; “when”: *F*(1, 48) = 0.338, *p* = .564]. There was also no relationship between BMI and time taken at encoding, suggesting that overweight participants were not simply less careful when committing events to memory, *F*(1, 114) = 0.30, *p* = .586.

Given the particular demand on spatial recall in the WWW task, it is possible that poor performance on the WWW task was driven solely by a problem with spatial memory. This was investigated by analysing the types of errors made in the WWW task. People with higher BMI made more errors in total; however, there was no relationship between BMI and number of “imprecision”, *F*(1, 48) = 0.107, *p* = .446. or “total inaccuracy”, *F*(1, 48) = 0.811, *p* = .372, errors. Instead, there was a significant relationship between BMI and number of “binding” errors, *F*(1, 48) = 5.278, *p* = .026. This suggests that the type of error driving the poorer performance in those with higher BMI was incomplete binding or integration of elements, rather than spatial inaccuracy per se.

### Discussion

The present results indicate that with increased BMI, young, otherwise healthy individuals show significant reduced capacity in episodic memory, as assessed by the Treasure-Hunt Task. Those with higher BMI showed impaired performance on spatial, temporal, and item memory, as well as the ability to bind these elements together into a single coherent representation (“what–where–when” memory). BMI explained significant additional variance in memory score for the individual “what”, “where”, and “when” tasks, but not the “WWW” task when variance due to age, gender, and years in education was controlled for.

Our findings could suggest that the structural and functional neural changes that have been demonstrated in those with elevated BMI may be accompanied by significantly reduced ability to form and/or retrieve episodic memories. Importantly, this effect is present in young, nondiabetic individuals. This adds to the growing data suggesting that the cognitive impairments that accompany obesity are present early in adult life and are not driven by diabetes. However, it should be noted that the sample assessed in this study were not screened for the many other conditions that are comorbid with obesity (such as hypertension and sleep apnoea) and also associated with cognitive deficits (Décary et al., 2000; Kilander et al., 1998). Furthermore, while the participants had never received a diagnosis of diabetes, this does not rule out the possibility that they had undiagnosed problems with insulin resistance. As such, it is possible that the memory effects reported here may be driven by conditions comorbid with obesity rather than adiposity itself.

To date, the association between obesity and memory has yet to be convincingly demonstrated in humans. Given the current findings, it may be that the failure of previous research in humans to reliably replicate the rodent findings of obesity-related memory deficits may have been attributable to the use of word-list paradigms. The study presented here used a nonverbal memory paradigm that required the integration of item, spatial location, and temporal order into a single coherent representation and in this way gets closer to the context-rich nature of episodic memory as it is used and experienced in everyday life. This study had a small sample size, and thus we must be cautious with interpretation, especially given the fact that variance explained by BMI dropped below significance once demographic factors were accounted for, which suggests that the effect may not be robust against variation due to extraneous factors. While there was no significant relationships between memory and these demographic factors, null results should be treated with caution when degrees of freedom are limited. Further research is necessary to establish whether the results of this study can be generalized to overweight individuals in general, and to episodic memory in everyday life. However, the possibility that there may be episodic memory deficits in overweight individuals is of major concern, especially given the growing evidence that episodic memory may have a considerable influence on feeding behaviour and appetite regulation (Robinson et al., 2013).

The idea that impaired cognitive performance in obesity may affect the regulation of consumption has been put forward by Gunstad and Sellbom (Sellbom & Gunstad, 2012) and Davidson and colleagues (Davidson, Kanoski, Walls, & Jarrard, 2005; Kanoski & Davidson, 2011). Both groups propose “vicious cycle” models of obesity and cognitive decline, in which cognitive impairments associated with obesity may impact learning, which in turn impacts ability to regulate weight. Davidson and colleagues (Davidson et al., 2014) hypothesize that the reason that memory for recent meals affects later consumption is that it acts as a negative feature stimulus that is informative about the likely postingestive sensory consequences of intake; that is, the presence of the memory is a predictive stimulus as to the physiological consequences of further consumption. Within their framework, memories have no special associative properties per se and therefore operate in the same way as conventional external stimuli when they are embedded in the set of associative relationships. If associative learning is disrupted, therefore, then the ability to form associations between memories and physiological outcomes is also disrupted. Given the present results, it is possible that obesity may also be related to problems with explicit episodic memory itself. These accounts are not mutually exclusive; it is likely that changes to hippocampal function would result in problems with *both* episodic memory and negative-pattern associative learning, making the resulting problems even more likely to cause issues with consumption regulation. Of course, such an account requires further empirical confirmation, and it is not clear to what degree the present findings can be applied to consumption memories.

#### Caveats and provisos

This is the first study to use the Treasure-Hunt Task, and as such there are as yet no established norms for this task, and its validity for assessing episodic memory across diverse populations is yet to be established. For this reason, further research is needed before we can conclude with confidence that the associations between BMI and memory seen in this small, cross-sectional study are generalizable to the general population in everyday life. In the present study the WWW what, where, and when elements differed not only in the information assessed but also in the retrieval support provided. Some tests required only recognition, whereas others provided fewer retrieval cues. It is therefore possible that different performances across these tasks were due in some degree to differences in the requirement for retrieval support rather than the ability to remember particular types of information. Furthermore, it is difficult to identify whether there is a specific deficit in memory integration, given the impairments on the individual elements of the memory. Future studies should control for retrieval support across the different tests and should assess integration independently of spatial memory. Moreover, it is possible that the fixed order of the task presentation may have impacted performance on different tests unevenly. As such, future studies should present these tests in a counterbalanced order.

Finally, this study has concentrated on the impact of excess weight on episodic memory. However, as reviewed in the introduction, obesity is associated with impairments in a range of cognitive functions, and it is likely that any impairment would not be specific to memory. Conversely, cognitive impairments have been indicated in a number of conditions comorbid with obesity (such as hypertension and sleep apnoea), ones that were not assessed in this study. As such, future research should investigate memory impairments in the context of other obesity-related conditions and cognitive abilities.

In summary, the current findings suggest that individuals with higher BMI may exhibit a deficit in episodic memory relative to lean controls. This study focused on a small cross-section of individuals. However, if generalizable, this finding is concerning, especially given evidence that memory may be an important factor in the regulation of consumption. Further research should investigate whether overweight individuals are less able to encode and/or retrieve meal memories, and whether this impacts later consumption.

### Supplemental material

Supplemental material is available via the “Supplemental” tab on the article's online page (http://dx.doi.org/10.1080/17470218.2015.1099163).

## ORCID

*Lucy G. Cheke* http://orcid.org/0000-0001-5588-7575
